# Age-Independent Clinical Outcome in Proximal Humeral Fractures: 2-Year Results Using the Example of a Precontoured Locking Plate

**DOI:** 10.3390/jcm11020408

**Published:** 2022-01-14

**Authors:** Rony-Orijit Dey Hazra, Johanna Illner, Karol Szewczyk, Mara Warnhoff, Alexander Ellwein, Robert Maximillian Blach, Helmut Lill, Gunnar Jensen

**Affiliations:** Department of Orthopedic and Trauma Surgery, Diakovere Friederikenstift and Henriettenstift, 30169 Hannover, Germany; Johanna.Illner@stud.mh-hannover.de (J.I.); Karol.Szewczyk@diakovere.de (K.S.); Mara.Warnhoff@diakovere.de (M.W.); Alexander.Ellwein@diakovere.de (A.E.); robmblach@gmail.com (R.M.B.); Helmut.Lill@diakovere.de (H.L.); Gunnar.Jensen@diakovere.de (G.J.)

**Keywords:** proximal humeral fracture, fracture in the elderly, precontoured locking plate, screw augmentation

## Abstract

Introduction: The optimal treatment strategy for the proximal humeral fracture (PHF) remains controversial. The debate is centered around the correct treatment strategy in the elderly patient population. The present study investigated whether age predicts the functional outcome of locking plate osteosynthesis for this fracture entity. Methods: A consecutive series of patients with surgically treated displaced PHF between 01/2017 and 01/2018 was retrospectively analyzed. Patients were treated by locking plate osteosynthesis. The cohort was divided into two groups: Group 1 (≥65 years) and Group 2 (<65 years). At the follow-up examination, the SSV, CMS, ASES, and Oxford Shoulder Score (OS), as well as a radiological follow-up, was obtained. The quality of fracture reduction is evaluated according to Schnetzke et al. Results: Of the 95 patients, 79 were followed up (83.1%). Group 1 consists of 42 patients (age range: 65–89 years, FU: 25 months) and Group 2 of 37 patients (28–64 years, FU: 24 months). The clinical results showed no significant differences between both groups: SSV 73.4 ± 23.4% (Group 1) vs. 80.5 ± 189% (Group 2). CMS: 79.4 ± 21 vs. 81.9 ± 16, ASES: 77.2 ± 20.4 vs. 77.5 ± 23.1, OS: 39.5 ± 9.1 vs. 40.8 ± 8.2; OS: 39.5 ± 9.1 vs. 40.8 ± 8.2. In the radiological follow-up, fractures healed in all cases. Furthermore, the quality of fracture reduction in both groups is comparable without significant differences. The revision rate was 9.5% in Group 1 vs. 16.2% in Group 2. Discussion: Both age groups show comparable functional outcomes and complication rates. Thus, the locking plate osteosynthesis can be used irrespective of patient age; the treatment decision should instead be based on fracture morphology and individual patient factors.

## 1. Introduction

The optimal treatment of proximal humeral fractures (PHF) remains controversial, although this fracture entity accounts for up to 4–5% of all fractures and is the third most common fracture type in the elderly [1,2]. With the aging of the global population and concomitant increase in osteoporosis cases, the incidence rate is expected to increase considerably [3]. While nonoperative management of PHFs has certain advantages, most epidemiological studies predict that surgical treatment rates will increase by up to 30% [4,5,6]. Reverse shoulder arthroplasty and the conventional locking plate are the most frequently used operative treatments [6,7]. Although the AO Foundation recommends an open reduction in the fracture and fixation with a locking plate, high failure rates have resulted in widespread skepticism regarding this approach [8,9,10,11,12]. Possible reasons for failure are poor fracture reduction, avascular necrosis, varus malunion, screw cutout, and unsatisfactory functional outcomes [8].

The high complication rate associated with locking plate osteosynthesis has led to technical modifications and the development of new procedures [13,14,15], including screw augmentation [13,16,17,18], medial calcar stabilization by bone grafting [19], carbon fiber-reinforced plates [13,20,21], and soft tissue-preserving approaches [22]. Our research group recently reported a failure rate of 9.3% and a mandatory reoperation rate of 15.6% using a standardized treatment algorithm (Table 1) [23]; however, there is little known about age-related differences in epidemiology, fracture morphology, and functional outcome. The critical limit for age-related fractures in various studies is 65 years [24,25]. The aim of this study was to investigate whether age influences the efficacy of locking plate osteosynthesis by comparing the functional outcomes of patients ≥65 years and <65 years. Patients were solely treated with conventional locking plates following a strict treatment algorithm at a level-1 trauma center with a focused unit on shoulder fractures. It was hypothesized that following a strict therapy algorithm, the functional outcome is independent of age with similar complication rates.

## 2. Materials and Methods

### 2.1. Case Selection

This was a retrospective study with a prospective follow-up component. The primary decision regarding the therapeutic strategy was based on our previously published treatment algorithm (Table 1) [13].

All cases of PHF treated between January 2017 and January 2018 at a level-1 trauma center were evaluated. A clinical examination was performed in all patients who underwent fracture fixation with the titanium alloy PHILOS plate (DePuy Synthes^®^; Synthes GmbH, Umkirch, Germany). Depending on fracture morphology, the surgeon had the choice of performing additional humeral head screw augmentation with polymethyl methacrylate cement or by double plating, as described in our previous work [13,26]. This study was carried out after consulting with the institutional ethics committee (Medizinische Hochschule Hannover, 8653_BO_S_2019) and in accordance with national legal requirements. All procedures that were performed complied with the ethical standards of the institutional and/or national research committee and with the 1975 Helsinki declaration and its later amendments or comparable ethical standards. Informed consent was obtained from all patients prior to their participation in the study. Inclusion criteria were as follows: (1) displaced, unilateral PHF; (2) age ≥ 18 years; (3) provided written, informed consent; (4) followed up for a minimum of 12 months; and (5) fixation with the titanium alloy PHILOS plate. Patients were excluded from the study if they had neurological disease with impairment of the upper extremity; dementia; psychiatric disease; language barrier; immunosuppression; death; were in poor condition after chemotherapy/radiotherapy; required supervision; or had muscular disease, bilateral PHF, posterior locked dislocation, poly-ether-ether-ketone plate osteosyntheses, or previous shoulder surgery. The study population was divided into 2 subgroups according to age: Group 1 (≥65 years) and Group 2 (<65 years). Preoperative patient-specific data such as age, sex, affected side, past medical history, use of medications, and concomitant fractures were noted. PHFs were preoperatively classified according to established criteria [27].

### 2.2. Surgical Approach and Postoperative Management

**Figure 1:**
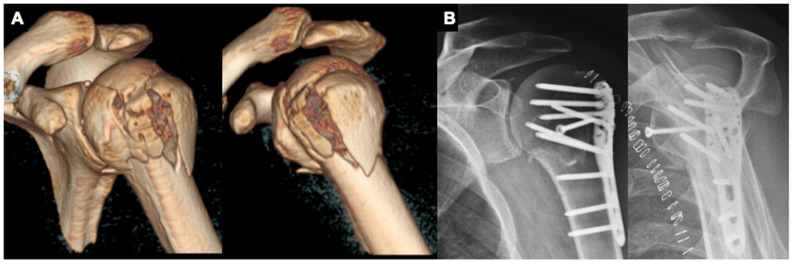
(**A**,**B**): 57-year-old male patient with a varus-impacted 4-part proximal humeral fracture with headsplit treated with a PHILOS plate (DePuy Synthes^®^, Umkirch, Germany) and additional free humeral head screw.

**Figure 2:**
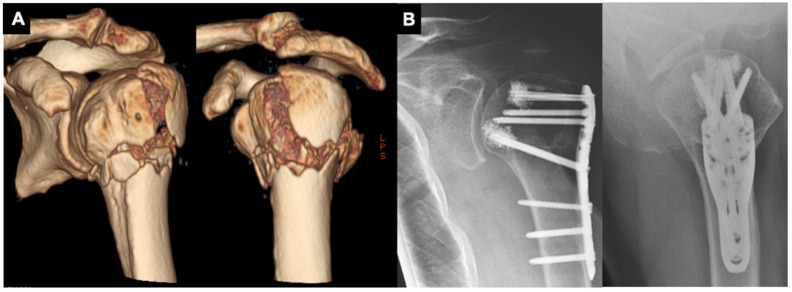
(**A**,**B**): 78-year-old male patient with a varus-impacted 3-part proximal humeral fracture treated with a PHILOS plate (DePuy Synthes^®^, Umkirch, Germany) and additional humeral head screw augmentation with PMMA trauma cement.

After being positioned in a modified thirty-degree “beach-chair” position, a soft tissue-preserving deltopectoral approach is performed. After careful preparation and preservation of the cephalic vein as well as the conjoint tendons, both anatomical landmarks are retracted medially (conjoint tendons) and laterally (cephalic vein). If further intraarticular repositioning is required, the rotator cuff interval is opened and an additional soft tissue tenodesis of the long head of the biceps is performed. Next, the reduction is performed with the help of an elevator or chisel, followed by positioning of the plate osteosynthesis to reduce the fracture with the help of it. The reduction is verified through fluoroscopy. If the reduction and plate positioning are adequate, all screws are inserted (Figure 1). If needed, sutures are positioned through the supraspinatus tendon and the subscapularis tendon to reduce the greater and lesser tuberosity, as well as to prevent secondary dislocation. In cases of poor bone quality, the surgeon has the choice of performing additional humeral head screw augmentation with polymethyl-methacrylate cement (Figure 2). Post-operatively, no arm sling is applied, and free passive and active motion was immediately allowed without weight-bearing for 6 weeks.

### 2.3. Functional and Radiologic Follow-Ups

All Patients underwent X-ray imaging on the second postoperative day. X-rays were performed in 2 planes (true anteroposterior [AP] and axial) at all times, and the quality of fracture reduction was assessed according to Schnetzke et al. [28]. Patients underwent one clinical and radiological follow-up at a minimum of 12 months after surgery. X-ray imaging was obtained at the follow-up unless previous radiographs had shown fracture consolidation. The neck-shaft angle (NSA) was measured using the standardized true AP view on the second day after surgery and at the follow-up. Radiographs were further analyzed to check for screw perforation, loss of fixation, fragment displacement, bony fracture consolidation, avascular necrosis of the humeral head and/or tuberosities, and implant loosening. The clinical examination included next to an assessment of the active range of motion, clinical scores, such as the age- and sex-adjusted Constant–Murley Score (CMS), the Oxford Shoulder Score (OS), the American Shoulder and Elbow Score (ASES), the Subjective Shoulder Value (SSV), as well as the time until return to sport/work was achieved. Complications were assessed. For this study’s setting, complications were defined as revision surgery or change of procedure from osteosynthesis to arthroplasty.

### 2.4. Statistical Analysis

Results were interpreted in a descriptive fashion and are presented as mean, standard deviation (SD), and range. Data were evaluated using Kolmogorov–Smirnoff normality testing followed by parametric analysis with Welch’s unpaired *t*-test for CMS and ASES scores and by nonparametric analysis with the Mann–Whitney U test for SSV and OS scores. Furthermore, the effect size was measured according to Cohen and graded to Gignac (weak d = 0.10; moderate d = 0.30; strong d = 0.50) [29,30]. The epidemiology of all PHFs was evaluated with the Chi-squared test, and subgroups were analyzed by a 2-way analysis of variance. *p* values < 0.05 were considered significant.

## 3. Results

During the observation period, 251 patients with 253 PHFs presented at our center. A total of 131 patients (132 PHFs) underwent primary fracture fixation of a displaced PHF with the titanium alloy PHILOS plate. In total, 36 patients were excluded because they did not meet the inclusion criteria. Among them, patients with: neurological disease with impairment of the upper extremity (*n* = 10); dementia (*n* = 6); psychiatric disease (*n* = 3); language barrier (*n* = 3); immunosuppression (*n* = 3); death (*n* = 3); were in poor condition after chemotherapy/radiotherapy (*n* = 2); required supervision (*n* = 2); or had muscular disease (*n* = 2), bilateral PHF (*n* = 1), posterior locked dislocation (*n* = 1), or poly-ether-ether-ketone plate osteosyntheses (*n* = 24). Additionally, 16 patients were unwilling to participate in the study, leaving 79 patients who underwent a follow-up examination.

### 3.1. Demographic and Clinical Characteristics of the Study Population

Group 1 (≥65 years) included 152 patients with a mean age of 76.8 ± 7.3 years. The distribution of fracture types in this group was as follows: three-part fracture, 43.4% (*n* = 66); four-part fracture, 32.2% (*n* = 49); two-part fracture, 7.9% (*n* = 12); isolated major tubercle fracture, 5.9% (*n* = 9); and dislocated shoulder fracture, 3.2% (*n* = 5) (Table 2). A headsplit component was present in 11 patients (7.2%). The following treatments were used: plate osteosynthesis, 57.2% (*n* = 87), of which 74 (48.7%) were with PHILOS plates and 13 (8.6%) were with polyetheretherketone (PEEK) plates; reverse arthroplasty, 21.7% (*n* = 33); conservative approach, 20.4% (*n* = 31); and nail osteosynthesis, 0.66% (*n* = 1). From the 74 patients treated in Group 1 using the titanium alloy plate, 25 did not meet the inclusion criteria and 7 patients did not want to participate, leading to a follow-up group of 42 patients (85.7%), with a mean age of 74.4 ± 6.6 years (range: 65–89 years) and mean follow-up time of 25 months (range: 19–29 months). The fracture types in this group were as follows: three-part fracture, 50% (*n* = 21); four-part fracture, 31% (*n* = 13); two-part fracture, 9.5% (*n* = 4); isolated major tubercle fracture, 4.8% (*n* = 2); and dislocated fracture, 4.8% (*n* = 2) (Table 2). The fractures were classified as varus-impacted in 15 cases, varus-distracted in 5 cases, valgus-impacted in 16 cases, and valgus-distracted in 5 cases, with 1 case of headsplit fracture. Cement augmentation of the humeral head screws was performed in 18/42 cases (43%).

Group 2 (<65 years) included 101 patients with a mean age of 53.0 ± 9.1 years. Fracture types in this group were as follows: four-part fracture, 35.6% (*n* = 36); three-part fracture, 27.7% (*n* = 28); isolated major tubercle fracture, 13.8% (*n* = 14); dislocated shoulder fracture, 8.9% (*n* = 9); and two-part fracture, 5.9% (*n* = 6). Seven patients (6.93%) presented with a headsplit component. The following treatments were used: plate osteosynthesis, 68.3% (*n* = 69), of which 58 (57.4%) were PHILOS plates and 11 (10.9%) were PEEK plates; conservative approach, 21.8% (*n* = 22); and reverse arthroplasty, 9.9% (*n* = 10).

Of the 58 patients treated in Group 2 using the titanium alloy plate, a total of 13 did not meet the inclusion criteria and 8 patients did not want to participate, leading to a follow-up group of 37 patients (82.2%), with a mean age of 52.9 ± 8.9 years (range: 29–65 years) and a mean follow-up time of 24 months (range: 14–29 months). Fracture distribution in this subgroup was as follows: four-part fracture, 56.7% (*n* = 18); three-part fracture, 27% (*n* = 10); two-part fracture, 10.8% (*n* = 4), isolated major tubercle fractures, 8% (*n* = 3); and dislocated fracture, 5.4% (*n* = 2). The fractures were classified as varus-impacted in 7 cases, varus-distracted in 5 cases, valgus-impacted in 19 cases, and valgus-distracted in 3 cases, with 3 cases of headsplit fracture. A total of 26 double plating procedures were performed in 1 of the 37 cases.

### 3.2. Functional Outcomes

**Figure 3:**
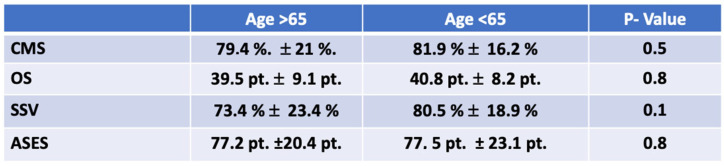
Clinical scores. Comparable clinical outcomes of patients over and under the age of 65 without any significant differences.

Comparing Group 1 (>65 years of age) to Group 2 (<65 years of age), the mean ± SD functional outcome scores/values were as follows: CMS, 79.4 ± 21.05 points (range: 35–100 points) in Group 1 as compared to a CMS of 81.9 ± 16.2 points (range: 37–100 points) in Group 2 (*p* = 0.5) with a weak effect size (d = 0.142); OS, 39.5 ± 9.1 points (range: 20–48 points) in Group 1 to an OS of 40.8 ± 8.2 points (range: 18–48 points) in Group 2 (*p* = 0.8) with a weak effect size (d = 0.015); SSV, 73.4% ± 23.4% (range: 20–100%) in Group 1 to an SSV of 80.5% ± 18.9% (range: 30–100%) in Group 2 (*p* = 0.1) with a strong effect size (d = 0.5); an ASES of 77.2 ± 20.4 points (range: 40–100 points) in Group 1 as compared to 77, 53 ± 23.14 points in Group 2 (range: 28.3–100 points) (*p* = 0.8) with a moderate effect size (d = 0.4) (Figure 3). In Group 1, the average time until returning to activities of daily living and sports was 65 ± 66/158 ± 147 days, and in Group 2, the times until return to work and sports were 105 ± 106 and 180 ± 163 days, respectively.

### 3.3. Radiological Findings

Table 3 summarizes the quality of fracture reduction in the postoperative imaging of all patients included in this study.

In Group 1, radiological follow-up was performed in 20/42 patients (47.6%) after a mean postoperative time of 25 months; 22 patients refused to undergo a follow-up X-ray examination. The fractures had healed in all cases. Secondary screw perforation was observed in 3/20 cases (15%) and fragment displacement in 2/20 cases (10%), both involving displacement of the greater tubercle. There was no case of loss of fixation. Average NSA was 127.1° ± 5.6° immediately after the surgery and 124.9° ± 5.1° at follow-up. Avascular necrosis was observed in 2/31 patients (6.4%).

In Group 2, 26/37 patients (70.3%) underwent radiological follow-up at a mean time of 24 months post-surgery; 11 patients refused to undergo a follow-up X-ray examination. The fractures had healed in all cases. Secondary screw perforation was observed in 4/26 cases (15.4%) and fragment displacement in 0/26 cases (0%). The average NSA was 122.8° ± 5.9° immediately after the surgery and 122.4° ± 6.6° at follow-up.

### 3.4. Clinical Complications

In Group 1, a total of two cases showed osteonecrosis of the humeral head and were treated using reverse shoulder arthroplasty. In the first case, a 70-year-old female with a valgus-distracted four-part fracture experienced necrosis 4 months after primary surgery. In the second case, an 81-year-old female with an initially valgus-impacted four-part fracture presented with necrosis of the humeral head 5 months after primary plate osteosynthesis, and treatment was also switched to reverse shoulder arthroplasty. Of the already mentioned three cases of screw perforation, two cases required revision surgery due to screw perforation; one case was in the cement-augmented humeral head screw subgroup, and the other was in the subgroup without cement.

In Group 2, a total of four patients suffered an avascular humeral head necrosis, leading to three reverse arthroplasties and one anatomical shoulder arthroplasty. In two cases, divergent from the treatment algorithm, humeral head-preserving reconstruction was performed even though both fractures had a humeral headsplit component. The other two cases showed a varus-distracted pattern with a severe commuted humeral head requiring allogeneic bone transplantation. Late infection was observed in a 53-year-old female with diabetes mellitus type 2 (on medication) who had a valgus-impacted four-part fracture and was initially treated by plate osteosynthesis; 19 months after the surgery, the patient requested removal of material, which was performed arthroscopically. Three weeks later, she suffered a joint infection that required multiple surgeries, leading to reverse shoulder arthroplasty. One case of early-onset infection in a 64-year-old female without relevant diseases in her medical history required multiple revision surgeries, which ultimately led to reverse shoulder arthroplasty.

## 4. Discussion

The most important finding of this study is that age had no influence on the functional outcome of locking plate osteosynthesis for the treatment of PHFs. Furthermore, complication rates were similar between patients ≥65 and <65 years of age (Groups 1 and 2, respectively) if the cases for surgery were carefully selected and treated according to a strict algorithm.

Age and fracture complexity play key roles in determining the optimal treatment strategy. Previous studies showed an age-dependent shift in the distribution of fractures from two-part to three- and four-part fractures [13,31,32], which are considered complex, difficult-to-treat entities [33]. This is consistent with the findings of this study illustrating that two-part fractures accounted for around 10% of all fracture types in both age groups and that three- and four-part fractures were the most common (Group 1: 50% and 31%, respectively; Group 2, 27% and 48%, respectively). Although four-part fractures were more common than three-part fractures in patients <65 years while the opposite trend was observed in patients ≥65 years, the overall frequency of complex fractures was similar in both age groups. However, based on the therapy algorithm treating head split fractures differently between both age groups, the age group under 65 seems to illustrate an increased fracture severity. Complications are among the greatest challenges in the surgical treatment of fractures with a precontoured locking plate in the elderly [8,34,35]. In contrast to a previous report in which complication rates for three- and four-part fractures were 39% and 45%, respectively [36], in this study population, the rates were 9.5% and 16.2%, respectively, and were similar between the two age groups. This is in line with previously published epidemiological data of this study group that showed a failure rate of 9.3% (*n* = 566) [23]. Interestingly, the complication rate associated with the precontoured locking plate was independent of age, which is contrary to the generally held view of therapeutic strategies for PHFs; this may be attributable to advances in medical technologies and standardized surgical techniques, such as screw augmentation [16,18,19,37], bone grafts for medial calcar stabilization [19], carbon fiber-reinforced plate systems [38], and soft tissue-preserving approaches [22]. In this study, 43% of patients ≥65 years treated with humeral head screws received partial cement augmentation (Figure 2). Patients with an augmentation recorded a humeral head screw perforation in one case. This is in line with previously published data showing excellent clinical outcomes with significantly lower rates of humeral head screw perforation as compared to humeral head screw augmentation using a conventional approach [26].

It has been asserted that the only two significant predictors of locking plate osteosynthesis failure are the skill level of the shoulder specialist and cigarette smoking [34]. This was supported by the finding that complications were related to an inadequate surgical technique in up to 40% of cases [8]. All procedures in this study were performed at a specialized shoulder and elbow unit at a level-1 trauma center, after discussion and under the supervision of certified senior shoulder surgeons. Thus, both age groups illustrated a similar quality of fracture reduction, without significant differences.

There was no statistically significant difference between the two age groups in terms of functional outcome. This is consistent with a previous study comparing PHFs treated with the PHILOS locking plate system that reported comparable ASES and OS scores at the 1-year follow-up in patients over and under 70 years of age [34]. In contrast to the previously mentioned study, this study also used subjective patient-reported outcome measures as well as objective measurements, such as CMS, with similar results. This is at odds with the increasing number of primary reverse shoulder arthroplasties performed for PHFs [6,7,39]. Based on a mathematical regression model of inpatients who were treated in the U.S. between 2004 and 2012, the use of reverse shoulder arthroplasty was projected to be 100% by 2032 because of demographic changes and a rising incidence of osteoporosis [6]. This estimate is contrary to that of a study in which radiographic loss of reductions was found in 6.7% of 252 PHFs treated with locking plates; a multivariate analysis identified osteoporosis, varus displacement, lack of medial support, and comminution but not age as independent risk factors [40]. Similarly, loss of reduction was observed in 6.4% (2/31) of our patients ≥65 years, amounting to a 2.9° change in NSA from the X-ray examination immediately following surgery to the follow-up examination. In patients <65 years, no loss of reduction was observed.

### Limitations

Patients ≥65 years were treated with reverse shoulder prostheses in cases of headsplit or comminuted fractures. Consequently, the treatment algorithm used in this study led to a significant increase in the rate of reverse shoulder arthroplasty in these patients as compared to the age group <65 years, in which the treatment of choice was a reconstruction attempt combined with locking plate osteosynthesis, which can lead to an algorithm-driven increased complexity in the age group under 65. Next, the retrospective study design, as well as the literature-based distinction between Group 1 (>65) and Group 2 (<65), is a limitation. Further, due to radiation exposure, the follow-up imaging was voluntary and led to 47.7% in Group 1 and 70.3% in Group 2, respectively.

## 5. Conclusions

Both age groups show comparable functional outcomes and complication rates, with the restriction of a therapy algorithm based on increased fracture severity in the age group under 65. However, focusing on the presented therapy algorithm, the locking plate osteosynthesis can be used irrespective of patient age; the treatment decision should instead be based on fracture morphology and individual patient factors. Further high-powered prospective multicenter trials focusing on additional age groups are needed to verify this preliminary data.

## Figures and Tables

**Table 1:** Therapy Algorithm. Nondisplaced fractures were conservatively treated [13].

Fracture Pattern	18–60 Years	61–70 Years	>70 Years
Greater tubercle	locking plate	locking plate	locking plate
2-part	locking plate/nail	locking plate	locking plate
3-part	locking plate	locking plate	locking plate
4-part	If possible locking plate/ anatomic arthroplasty	If possible locking plate/ reverse arthroplasty	If possible locking plate/ reverse arthroplasty
Headsplit/comminuted fractures	If possible locking plate/ anatomic arthroplasty	reverse arthroplasty	reverse arthroplasty

**Table 2:** Age, gender, and fracture pattern distribution of the age groups over and under 65 years treated with a locking plate. Whilst there is no significant difference in the allocation of 2-part fractures (*p* = 0.63), there is a significant increase in 4-part fractures (*p* ≤ 0.001) in the age group over 65. R stands for Range in this table and * for statistical significance.

	>65	<65	*p*-Value
Age	74.4 ± 6.6 (R: 65–89)	52.9 ± 8.9 (R: 29–65)	*p* ≤ 0.001 *
Male: Female	10:32	17:20	*p* = 0.007 *
2-part fracture	9.5%	11%	*p* = 0.63
3-part fracture	50%	27%	*p* ≤ 0.001 *
4-part fracture	31%	56.7%	*p* ≤ 0.001 *
Operating time	73 ± 31 min	79 ± 30 min	*p* = 0.34

**Table 3:** Quality of fracture reduction according to Schnetzke et al. Regarding the quality of fracture reduction, both groups are comparable without significant differences.

Quality of Fracture Reduction	>65	<65	
Head-shaft displacement			
Anatomical	10 (24%)	11 (30%)	
Acceptable	17 (40%)	17 (46%)	
Anatomical or Acceptable	27 (64%)	28 (76%)	
Malreduction	15 (36%)	9 (24%)	
Head-shaft alignment			
Normal	35 (83%)	29 (78%)	
Acceptable	7 (16%)	4 (11%)	
Anatomical or Acceptable	42 (100%)	33 (89%)	
Malreduction	0	4 (11%)	
Tuberosity proximal migration			
Anatomical	33 (79%)	24 (65%)	
Acceptable	5 (12%)	12 (32%)	
Anatomical or Acceptable	38 (91%)	36 (97%)	
Malreduction	4 (9%)	1 (3%)	
Overall quality of reduction			***p*-value**
Anatomical	7 (17%)	6 (16%)	0.9 (NS)
Acceptable	17 (40%)	20 (54%)	0.2 (NS)
Anatomical or acceptable	24 (57%)	26 (70%)	0.2 (NS)
Malreduction	18 (43%)	11 (30%)	0.2 (NS)
